# Effectiveness of F18^+^ Fimbrial Antigens Released by a Novel Autolyzed *Salmonella* Expression System as a Vaccine Candidate against Lethal F18^+^ STEC Infection

**DOI:** 10.3389/fmicb.2016.01835

**Published:** 2016-11-22

**Authors:** Gayeon Won, John H. Lee

**Affiliations:** College of Veterinary Medicine, Chonbuk National UniversityIksan, South Korea

**Keywords:** F18 fimbriae, STEC, porcine edema disease, inactivated *Salmonella* delivery system

## Abstract

Porcine edema disease (ED) caused by Shiga toxin 2e producing *Escherichia coli* expressing F18ab^+^ fimbriae (F18ab^+^STEC) frequently occurs in post-weaned piglets, resulting in a significant economic loss in swine industries worldwide. In the present study, we proposed an efficient prevention scheme against ED in which the attenuated *Salmonella* Typhimurium inactivated by the *E*-mediated cell lysis to deliver target antigens, FedF and FedA, which function in fimbrial-mediated adhesion and as a major subunit of F18ab+fimbriae, respectively. The co-expression of FedA and FedF protein with outer membrane protein A signal peptide was confirmed in the resultant strains JOL1460 and JOL1464 by immunoblot analysis. Immunization with the candidate strains in mice led to the significant generation of immunoglobulin (Ig) G, specific to both antigens and secretory IgA specific to FedF (*P* < 0.05). The titers of IgG isotypes, IgG1 and IgG2a, used as markers for T-helpers (Th)-2 and Th-1lymphocytes, respectively, also significantly increased in the immunized group (*P* < 0.05). The increase in CD3^+^CD4^+^ T lymphocyte subpopulation and *in vitro* proliferative activity was observed in *in vivo* stimulated splenocytes, which indicated the immunostimulatory effect of the candidate strains. Moreover, the immunized mice were completely protected from a lethal challenge against wild-type F18^+^STEC whereas 28% of mice died in the non-immunized group. This study demonstrated that the inactivated *Salmonella* system could efficiently release FedF and FedA and induce robust immune responses specific to the target antigens, which is sufficient to protect the mice from the lethal challenge.

## Introduction

Fimbrial adhesin F18 is frequently observed in enterotoxigenic *Escherichia coli* (ETEC) and Shiga toxin 2e producing *E. coli* (STEC) strains that cause diarrhea and edema disease (ED) in post-weaned piglets, respectively, resulting in a significant burden on swine industries worldwide (Nagy et al., 1997). The F18 fimbriated *E. coli* have two major antigenic variants, F18ab and F18ac, which mediate the intestinal colonization (Rippinger et al., 1995). Most F18ab^+^STEC isolates are associated with Shiga toxin 2e causing ED, while F18ac^+^ETEC produces enterotoxin ST-I (Nagy et al., 1997). The mechanism of pathogenesis in ED causes high mortality in affected piglets. The STEC colonization in the small intestine is initiated by F18ab^+^ fimbriae-mediated adherence to the receptor on the brush border of the porcine enterocyte (Imberechts et al., 1992b). Following colonization, Shiga toxin released into the vascular endothelium induces inhibition of protein synthesis and cell death, causing submucosal edema and neurologic symptoms in weaned piglets (Imberechts et al., 1992a).

Due to the significant burden of ED in weaned piglets worldwide, different strategies have been attempted to develop efficacious vaccine candidates against ED (Melkebeek et al., 2013). Stx2e-related proteins are typically used as a target antigen in the genetically engineered vaccine constructions (Gordon et al., 1992; Bosworth et al., 1996; Johansen et al., 1997; Matsui et al., 2009) Given the role of F18ab+ fimbriae in adhesion and their highly conserved structures (Wizemann et al., 1999), the F18ab+fimbriae gene cluster could be utilized as a potential vaccine candidate against the disease. F18 fimbriae consist of a major subunit, FedA and two minor subunits, FedE and FedF (Smeds et al., 2003). The minor protein, FedF, which is the conserved region in the F18ab^+^ fimbriae (Smeds et al., 2001), mainly functions in fimbrial-mediated adhesion of the STEC (Smeds et al., 2001). The backbone of the F18 fimbriae, FedA is also known to have a potent antigenic property (Bosworth et al., 1998).

The Gram-negative bacteria inactivated by the lysis gene *E* that is essential for the lytic function of ϕX174-Coliphage have been efficiently used as a homologous vaccine and in heterologous antigen delivery (Ebensen et al., 2004; Tabrizi et al., 2004; Walcher et al., 2004). The *E* gene inhibits the phospho-MurNAc-pentapeptide translocase in peptidoglycan biosynthesis, resulting in lysis of the bacterial cell wall (Bugg et al., 2006). Since the *E* gene-mediated lysis does not disrupt antigenic surface components containing lipopolysaccharide and peptidoglycan, the lysed bacteria have induced mucosal, humoral and cellular immune responses against target antigens (Haslberger et al., 2000). Specifically, *Salmonella enterica* serotypes inactivated by the lysis gene *E* such as *S*. Enteritidis (Jawale and Lee, 2014) and *S*. Typhi (Wen et al., 2012) have been successfully prepared for a foreign antigen carrier. Further, the lysed cells can be recognized and processed by dendritic cells (Kudela et al., 2005), promoting protective efficacy (Mayr et al., 2005; Chen et al., 2014; Zhu et al., 2015). In addition to these immuno-stimulatory properties of the lysed cell, the capacity of the cells being efficiently engulfed by antigen presenting cells also allows providing their intrinsic adjuvant property to the immunized host when the cells lysed by the expression of the gene *E* are utilized to deliver heterologous antigens (Ebensen et al., 2004; Walcher et al., 2004).

In this study, we constructed inactivated *S.* Typhimurium strains expressing FedF and FedA antigens as vaccine candidates against ED. In our approach, the *fedF* and *fedA* genes, respectively, were inserted into a heterologous protein delivery site of the recombinant plasmid pJHL184 carrying the lysis gene *E* cassette (Hur and Lee, 2015) and the aspartate β–semialdehyde dehydrogenase (*asd*) gene. The *asd* gene which synthesize diaminopimelic acid (DAP) has been used as non-antibiotic selective marker for the vaccine candidates (Garmory et al., 2005). Further, the balanced-lethal system based on the *asd* gene was applied to maintain the stability of the ghost plasmid in an attenuated *Salmonella* (Galán et al., 1990). The plasmids harboring the genes encoding the target protein were individually transformed into attenuated *Δasd Δlon ΔcpxR S.* Typhimurium strain JOL912. The ATP-dependent protease Lon encoded by *lon* gene regulate *Salmonella* pathogenicity island (SPI)-1 by controlling the expression of invasion genes essential for systemic infection (Takaya et al., 2003). The signaling pathway Cpx system encoded by *cpxR* also relevant to the expression of the SPI-1 genes maintain the stability of cell envelopes and biosynthesis of P pili (Kenyon et al., 2002). In the previous study, *S.* Typhimurium mutant was constructed by deletion of these two genes related to virulence characteristics of *S.* Typhimurium, resulting in induction of significant pathogenicity attenuation of the strain (Kim et al., 2009). Under the optimal condition, the activation of the lysis gene *E* stringently regulated by convergent promoters simultaneously induced the programmed lysis of the *Salmonella* strain and expression of the target antigens (Jawale et al., 2014). Protective immunogenicity was evaluated in mice immunized with a combination of the inactivated *S*. Typhimurium strains releasing FedF and FedA, respectively. Further, we determined which fimbriae protein predominantly elicits the immune responses in the animal model.

## Materials and Methods

### Bacterial Strains and Growth Conditions

The bacterial strains and plasmids used in this study are described in **Table 1**. The *asd* deleted *S.* Typhimurium mutant were cultivated in Luria-Bertani medium or on Luria-Bertani agar plates and grown at 37°C with DAP (Sigma-Aldrich, St. Louis, MO, USA). The bacterial strains harboring the ghost gene cassette were grown on NB agar supplemented with 0.2% L-arabinose.

**Table 1:** Bacterial strains, plasmids used in this study.

Strain, plasmid	Description	Reference
**Bacterial strains**		
*E. coli*		
BL21(DE3)pLysS	F^-^ *ompT hsdSB* (rB^-^ mB^-^) *dcm gal λ*(DE3) pLysS Cmr	Promega
JOL232	F^-^ λ^-^ ϕ80 Δ(*lacZYA-argF*) *endA1 recA1 hadR17 deoR thi-1 glnV44 gyrA96 relA1 ΔasdA4*	Lab stock
JOL505	Wild-type LT^+^, K99^+^, F6^+^, F18^+^, *stx_2_*^+^, *stx_2e_*^+^ STEC isolate from pig	Lab stock
JOL654	Wild-type LT^+^, F18^+^, STa^+^, *stx_2_*^+^, *stx_2e_*^+^ STEC isolate from pig	Lab stock
***S*. Typhimurium**		
JOL911	*bbblon ΔcpxR*, a derivative of *S*. Typhimurium	Hur and Lee, 2010
JOL912	*Δlon ΔcpxR Δasd*, a derivative of *S*. Typhimurium	Hur and Lee, 2011
JOL 1400	JOL912 harboring pJHL184	This study
JOL 1460	JOL912 harboring pJHL184-*FedF*	This study
JOL 1464	JOL912 harboring pJHL184-*FedA*	This study
**Plasmids**		
pET28a	IPTG-inducible expression vector; Km^r^	Novagen
pET28a-*_FedF_*	pET28a derivative containing *_FedF_*	This study
pET28a-*_FedA_*	pET28a derivative containing *_FedA_*	
pJHL184	*asd*^+^ vector, pBR ori plasmid carrying ss *ompA*/His_6_, multiple cloning site, cI857/λPR promoter, araC P_araBAD_, *phi*X174 lysis gene *E*	Hur and Lee, 2015
pJHL184-*_FedF_*	pJHL184 harboring *_FedF_* gene	This study
pJHL184-*_FedA_*	pJHL184 harboring *_FedA_* gene	This study

### Production and Expression

The 846 and 450 bp DNA fragments encoding FedF and FedA proteins, respectively, were amplified in JOL505 using polymerase chain reaction (PCR) with primer pairs (FedA_F:5′-ccgcgaattccagcaaggggatgttaaat-3′ and FedA_R: 5′-ccgcaagcttga tgattacttgtaagta-3′, FedF_F:5′-ccgcgaattcgcgtctactctacaagta-3′ and FedF_R:5′-ccgcaagcttttactgtatctcgaaaacaa-3′). The PCR products digested by EcoRI and HindIII were cloned into the overexpression plasmid pET28a, producing pET28a-*fedF* and pET28a-*fedA*, respectively. Each plasmid was transformed into BL21 (DE3) pLysS (Jawale and Lee, 2014). Briefly, the N-terminally 6x-His-tagged FedF or FedA expressed in each *E. coli* BL21 strain was purified following the protocol previously described (Jawale and Lee, 2014). The concentration of purified protein was quantified using Bradford reagent (Bio-Rad Laboratories, Hercules, CA, USA). To insert *fedF* and *fedA* individually into the pJHL184, the fimbrial gene fragments originating from pET28a-*fedF* and pET28a-*fedA* were individually subcloned into EcoRI/HindIII-digested pJHL184 to generate pJHL184-*fedF* and pJHL184-*fedA*, respectively. The resultant plasmids were initially introduced into *Δasd E. coli* χ6212 (JOL232) to reduce instability of the plasmids in the balanced lethal system (Galán et al., 1990), and then the plasmids were electroporated into the Δ*asd*Δ*lon*Δ*cpxR S*. Typhimurium strain JOL912 which was prepared by deletion of the *asd* gene in Δ*cpxR* Δ*lon* S. Typhimurium strain JOL911 using allelic exchange method (Hur and Lee, 2010). The transformants were designated JOL1460 for the strain carrying FedF and JOL1464 for the strain carrying FedA. JOL912 harboring only the ghost plasmid pJHL184, JOL1400 was constructed following the process and used as the empty vector control.

### Inactivation Process

The JOL1460 and JOL1464 strains were inoculated into 200ml of nutrient broth (NB) under the lysis gene *E* repressing condition containing 0.2% L-arabinose and were incubated at 28°C with slow agitation. When the cultures reached logarithmic phase, the cells were harvested, and then washed three times with NB to eliminate arabinose. The cells were resuspended in 100 ml of NB and incubated at 42°C for 48 h with 200 rpm agitation to induce *E* gene-mediated lysis. The lysed cells were collected by centrifugation at 13,000 rpm for 20 min after the lysis process and washed twice with autoclaved phosphate-buffered saline (PBS) (pH 7.4). The pellets were stored at -70°C until further use. The morphological alteration of the lysed JOL1460 and JOL1464 was visualized by using scanning electron microscope (SEM) as previously described (Jawale et al., 2014).

### *In vitro* Fimbrial Protein Expression

Western blot analysis was performed to validate the expression of the individual recombinant heterologous antigens, FedF and FedA fused to downstream of the six-histidine tag of the pJHL184 from the inactivated *Salmonella* carrier. Following a published protocol (Jawale and Lee, 2014), the lysed cells of JOL1460 and JOL1464 were prepared. The lysed samples were separated by sodium dodecyl sulfate-polyacrylamide gel electrophoresis (SDS-PAGE) on 15% gels. Subsequently, the proteins separated on the gel were electrotransferred laterally onto membranes of polyvinylidene fluoride (Millipore, Billerica, MA, USA) and blocked with 3% bovine serum albumin. The protein expressed in the *S.* Typhimurium ghosts was detected using a primary anti-His antibody (1:5,000) and secondary an HRP-labeled anti-mouse IgG (1:8,000). The immunoreactive bands were developed by the West-OneTM Western Blot Detection System (iNTRON, KOR).

### Experimental Animals and Immunization Procedure

All experimental and animal management procedures described in this study were approved (CBNU2015-00085) by The Chonbuk National University Animal Ethics Committee in accordance with the guidelines of the Korean Council on Animal Care. The thirty 5-week-old female BALB/c mice which have been widely used to assess the immunogenicity of bacterial vaccine (Lauvau et al., 2001), were randomly divided into two groups. The mice in group A were injected intramuscularly with 1.0 × 10^8^ of mixture formula of the lysed JOL1460 and JOL1464 cells in 100 μl of sterile PBS at week 0 and week 2, respectively, following the previously published protocol (Hur and Lee, 2015). Non-immunized mice in group B were intramuscularly inoculated with 100 μl of sterile PBS. At week 0, 2, 4, and 6 post-immunization (PI), blood samples were collected from the mice via the infraorbital vein to evaluate immunoglobulin (Ig) G, G1, and G2a. To assess secretory IgA (sIgA) produced in the mice, vaginal washes were obtained from the mice using sterile PBS. All samples were kept at -70°C until used.

### Humoral Immune Responses

To evaluate humoral immunity elicited by the immunization, the presence of total IgG, IgG1, IgG2a, and sIgA antibodies against FedF and FedA fimbrial antigens, respectively, in sera and vaginal washes was determined by indirect enzyme-linked immunosorbent assay (ELISA) as previously reported (Hur and Lee, 2015). Purified FedF and FedA proteins (0.5 μg/ml) were used as coating antigens. The final concentration of serum IgG and sIgA were analyzed by a standard curve of purified mouse immunoglobulins (Southern Biotechnology, Birmingham, AL, USA). The ratio of IgG2a to IgG1 isotypes was determined by dividing values of optical density (OD) at 470 nm for IgG2a by the OD values for IgG1.

### Flow Cytometry

An alteration in T cell subsets of the immunized mice was assessed using fluorescence-activated cell sorting (FACS) (Jawale and Lee, 2014). Spleen cells were isolated from the immunized and non-immunized mice at day 7 PI and 1 × 10^6^ of the cells were seeded into a 96-well cell culture plate. The splenic cells were stained with the surface markers containing anti-mouse CD3a-PE and anti-mouse CD4-perCP-vio700 (Miltenyi Biotec, Bergisch Gladbach, Germany). After the stained cells were washed twice in FACS buffer (Miltenyi Biotec, Bergisch Gladbach, Germany), the stained CD3+ and CD3+CD4+ splenic cells were sorted with an MACSQuant^®^ Analyzer (Miltenyi Biotec, Bergisch Gladbach, Germany). The altered FACS profiles of CD3+ and CD3+CD4+ splenic T cell subsets in the immunized mice were analyzed in comparison to those from non-immunized mice.

### Cell Proliferation Assay

The magnitude of splenocyte proliferation following *in vitro* antigen stimulation were assessed by incorporation of MTT (3-(4,5-dimethylthiazol-2-yl)-2,5-diphenyltetrazolium bromide) which is only converted to blue formazan dye by actively proliferating cells (Denizot and Lang, 1986). Splenocytes were isolated from the immunized and non-immunized groups at week 2 PI. 1 × 10^6^ of the splenic cells were stimulated with 300 ng/ml of the purified FedF or FedA protein in triplicate, and then placed in a humidified 37°C, 5% CO_2_ incubator for 48 h. After incubation for 48 h, 1 mg/ml of MTT was added to the stimulated cells, and the mixed culture was incubated for 4 h at 37°C. The MTT solution was converted into blue formazan dye only when the stimulated cells are actively proliferating. After the incubation, the resulting formazan precipitation was dissolved in 100 μl dimethyl sulfoxide (DMSO). The absorbance spectrum at 490 nm of MTT formazan solutions was measured by a spectrophotometer.

### Cytokine Assay in *In vitro* Stimulated Splenocytes

To evaluate production of immunomodulatory cytokines elicited by the vaccine candidates, expression of IL-4 and IFN-γ mRNA secreted by T-helper-2 (Th-2) (Swain et al., 1990) and Th-1 clones (Scott, 1991), respectively, were measured in the splenocytes pulsed with each antigen. Splenocytes were aseptically isolated from the immunized mice and the non-immunized mice at week 2 PI. 1 × 10^6^ of splenocytes were stimulated with 500 ng/ml of either FedF or FedA antigens in a 96-well plate and were incubated with RPMI 1640 medium (GIBCO, cat. no.11875093) containing 5% FCS (GIBCO, cat. no. 10099141) at 37°C in a 5% CO_2_ incubator for 48 h. Total RNA from the stimulated cell cultures was extracted using GeneAll^®^ Hybrid-RTM (GeneAll Biotechnology, Seoul, Korea) and was converted into cDNA with the ReverTra Ace^®^ qPCR RT Kit (FSQ-101, TOYOBO, Japan). The level of IL-4 and IFN-γ mRNA expression was determined by real-time reverse transcription-polymerase chain reaction (RT-PCR) with SYBR^®^ Green Realtime PCR Master Mix (QPK-201, TOYOBO, Japan) following the manufacturer’s instructions. The primer sets were derived from the conserved regions of mouse β-actin (as an internal standard), IL-4 and IFN-γ (Overbergh et al., 1999). The relative mRNA quantification of each cytokine was analyzed using the threshold method, using Δ*C*_T_ values calculated based on the internal standard. The relative fold change of the cytokines expressed in the stimulated cells was presented as 2^-(ΔΔ^*^C^*^T)^, compared to those of the non-immunized group (Schmittgen and Livak, 2008).

### Challenge Scheme

BALB/C mice did not show any symptoms of ED when they are orally challenged with a wild-type F18ab+STEC strain, JOL654, even though natural transmission of ED occurs via an oral route. Instead, we found that intraperitoneal injection of the challenge strain caused similar symptoms of ED in the mice. The median lethal dose of JOL654 in mice (LD50, 2 × 10^7^ CFU) was evaluated using the Reed–Muench method (Reed and Muench, 1938). At 4 weeks after the last immunization, both the immunized and non-immunized groups were intraperitoneally injected with 100 μl of JOL654, which contained a titer of 2 × 10^7^ CFU. Mortality, clinical sign, and body weight were monitored in the challenged mice during the week after inoculation.

### Statistical Analysis

Non-parametric Mann–Whitney test was applied to evaluate the difference in immune responses elicited by the immunized and non-immunized groups. All data are expressed as means ± standard deviation. Differences were considered statistically significant when *P*-values were ≤ 0.05.

## Results

### Generation of *S*. Typhimurium Lysed by *E* Gene Expressing the Fimbrial Proteins

**FIGURE 1:**
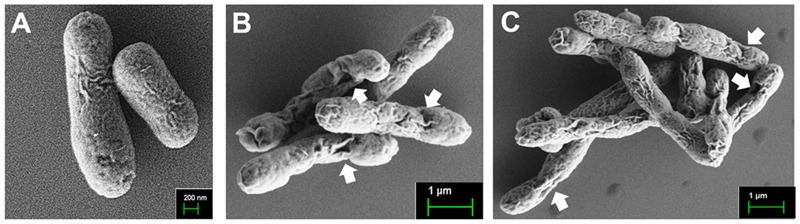
**Scanning electron microscopic (SEM) images of the lysed *S*. Typhimurium delivering FedF or FedA. (A)** Intact JOL1400 cells before lysis. **(B)** JOL1460 ghost cells **(C)** JOL1464 ghost cell. The arrows indicate the lysis transmembrane runnels.

**FIGURE 2:**
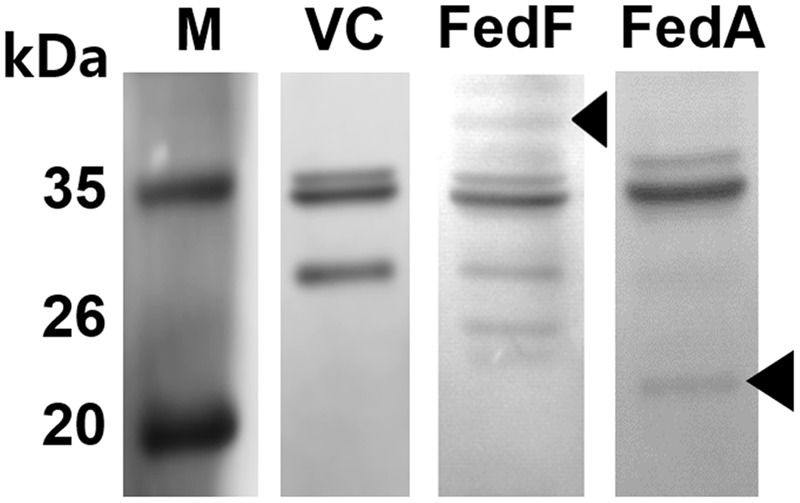
**Western blot analysis of FedF and FedA antigen expressed in JOL1460 and JOL1464 strains, respectively.** The proteins expressed and secreted by the strains were observed by immunoblotting, respectively. The arrows in lane FedF and FedA indicate ∼40 kDa and ∼24 kDa bands, representing the predicted size for FedF and FedA, respectively, fused with OmpA. JOL1400, pJHL184 electroporated into JOL912 was used as a vector control. Lane M, size marker; lane VC, vector control; lane FedF, a pellet of JOL1460; lane FedA, a pellet of JOL1464.

The *fedF* and *fedA* genes were each introduced into the heterologous antigen delivery site in the plasmid pJHL184. The resultant plasmids, pJHL184-*fedF* and pJHL184-*fedA* were individually transformed into the attenuated *S*. Typhimurium strain JOL912 to construct JOL1460 and JOL1464, respectively. The convergent promoter components induced expression of the *E* gene in pJHL184 under a temperature increase of up to 42°C and the removal of 0.2% L-arabinose. After 48 h at 42°C, the magnitude of lysis was determined by counting the number of viable cells plated from the culture of JOL1460 and JOL1464 grown at 42°C. No viable colony was detected on the LB plate supplemented with arabinose after overnight incubation at 27°C. Further, the transmembrane tunnel generated on the surface of lysed JOL1460 and JOL1464 was identified by the scanning electron microscopy (SEM) techniques. The cell surface appeared to be partially collapsed by releasing cytoplasmic contents through the pores on the cell membrane (**Figures 1B,C**) compared to the morphology of normal cells (**Figure 1A**). While the *E* gene-mediated lysis was activated, secretion of the fimbrial proteins into the cytoplasmic space was initiated. Subsequently, the proteins fused with the *ompA* signal sequence were secreted and stayed in the lysed *Salmonella*. Each fimbrial protein expressed in JOL1460and JOL1464 was validated by immunoblot analysis. The protein fused to OmpA expressed were detected at ∼40 kDa for FedF, and ∼24 kDa for FedA in the pellet of JOL1460 and JOL1464, respectively (**Figure 2**).

### Antibody Generation

**FIGURE 3:**
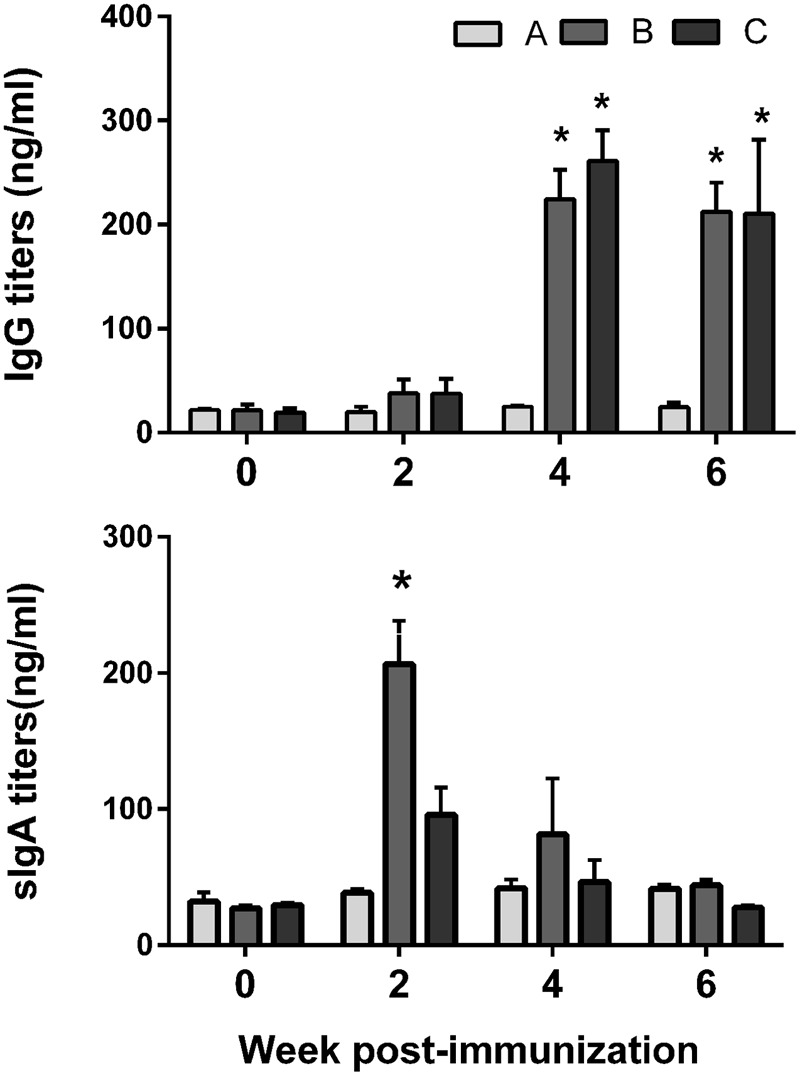
**Humoral immune responses specific to the fimbriae proteins elicited by immunization with JOL1460 and JOL1464 in the mice.** Data are the means for all mice in each group (*n* = 10). Group A, antibodies elicited in the non-immunized mice; B, antibodies specific to FedF elicited by the immunization in the mice; C, antibodies specific to FedA elicited by the immunization in the mice. Error bars indicate standard deviation (SD). PI, post-immunization; ^∗^*P* < 0.05 (vs. group A).

**FIGURE 4:**
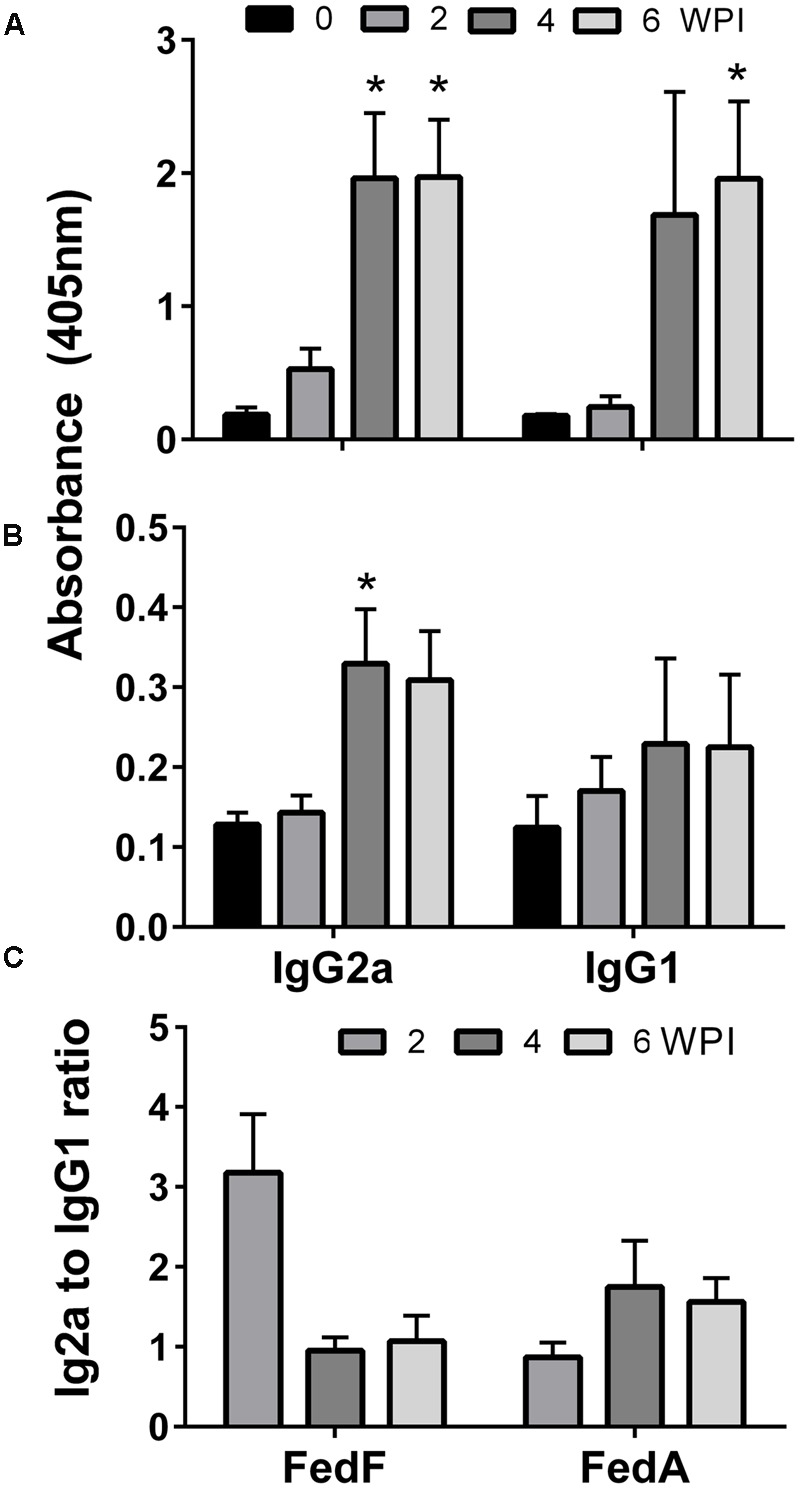
**The levels of two IgG isotypes, IgG1 and IgG2a, elicited by immunization with JOL1460 and JOL1464.** The values indicate the means of optical density at 450 nm in serum collected from immunized mice (*n* = 10). **(A)** IgG isotype antibodies specific to FedF; **(B)** IgG isotype antibodies specific to FedA; **(C)** IgG1/IgG2a ratios.

The titers of serum IgG and sIgA antibodies against FedF and FedA antigens were quantified in serum and vaginal wash samples, respectively. FedF-specific IgG and sIgA was markedly elevated (*P* < 0.05), whereas the IgG level specific to FedA was only significantly increased in mice at week 4 and 6 PI (*P* < 0.05) (**Figure 3**). At week 4 PI, the level of IgG against both FedF and FedA increased approximately 11 and 12.6 times, respectively, compared to levels in the non-immunized mice (**Figure 3**). The immunized mice generated 12.8 times more sIgA antibodies against FedF compared to those of the control at week 2 PI. The titers of serum IgG isotypes, IgG2a and IgG1, indicating activation of T helper (Th)-1 and Th-2 cells, respectively, were measured in the mice. The titer of IgG isotypes was presented as the OD values at 405 nm. The level of both IgG isotypes specific to FedF was nearly 10 times higher than those specific to FedA from week 4 to week 6 PI (**Figures 4A,B**). Additionally, the ratio of IgG2a to IgG1 was evaluated to elucidate relative contribution of Th-2 and Th-1 cells, respectively, to the immune responses. At week 4 and 6 PI, the ratio of IgG2a to IgG1 in the mice ranged from 0.9 to 1.76, which indicated that balanced Th-1 and Th-2-related immune responses were induced in the immunized mice (**Figure 4C**).

### T Cell-Mediated Immune Responses

**FIGURE 5:**
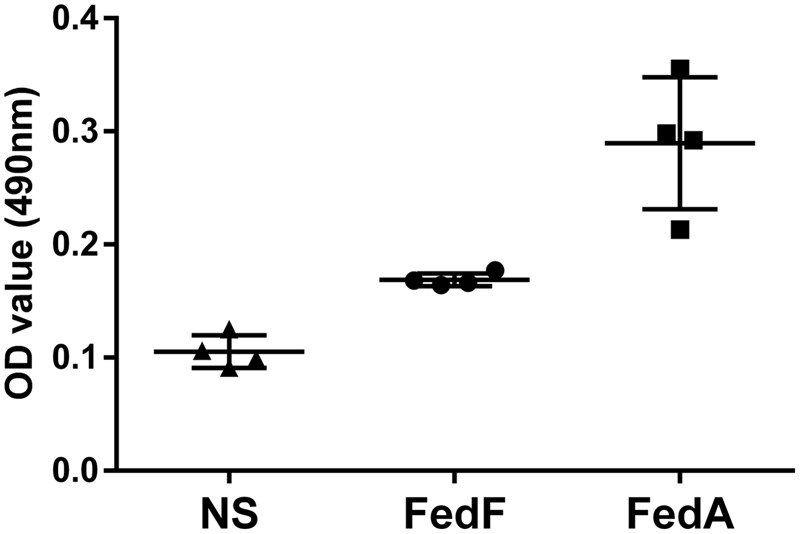
**Optical density (OD) values of splenic T cells from the immunized group (*n* = 4) stimulated with either FedF or FedA protein using the MTT assay.** Each point including triangle, circle and square represents the OD values obtained from the sample. The bars indicate the mean absorbance values in each group. Error bars indicate SD. NS, non-stimulated splenic T cell from the non-immunized group.

**FIGURE 6:**
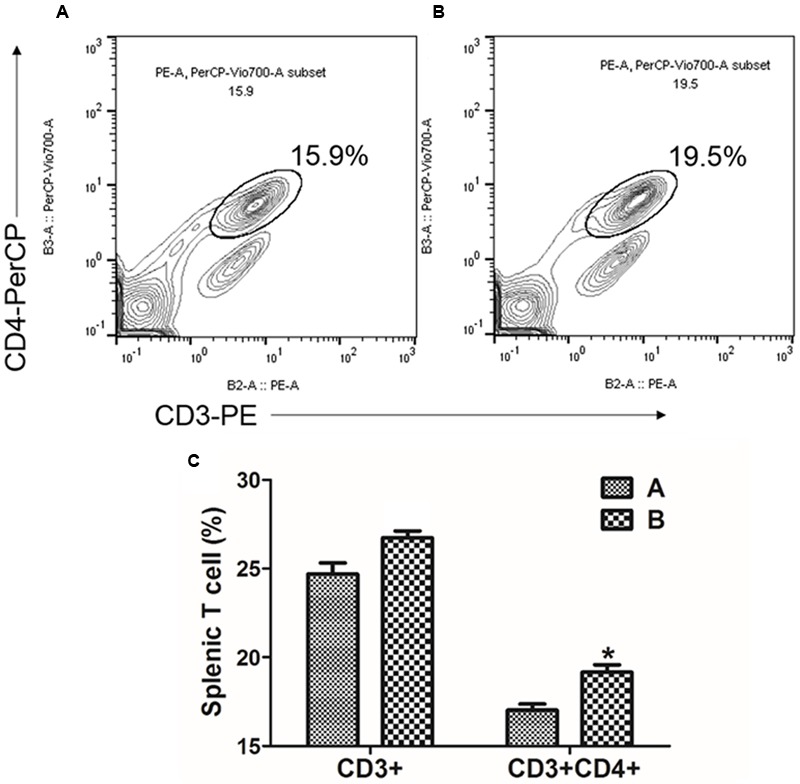
**Flow cytometric analysis for CD3+CD4+ splenic T lymphocyte population.** Representative flow cytometry scatter dot plots for CD3+, CD3+CD4+ splenic T cell populations of **(A)** the non-immunized mice (*n* = 4) and **(B)** the immunized mice (*n* = 4). The subpopulations are expressed as a percentage of the gated cells. **(C)** Change in T cell subpopulation in JOL1454 immunized mice. Group A, non-immunized mice; group B, immunized mice ^∗^*P* < 0.05 (vs. group A).

The MTT cell proliferation assay, the splenocytes stimulated *in vitro* with either FedA or FedF showed enhanced formazan absorbance values at 490 nm following the addition of MTT solution into the cell cultures (**Figure 5**). The absorbance value in FedF- and FedA-stimulated cells was augmented 1.53 ± 0.01 and 2.63 ± 0.08 times, respectively, in the comparison with those of the non-immunized mice at week 2 PI. The changes in T cell subsets was evaluated by detecting CD3^+^ and CD3^+^CD4^+^ surface marker expression in splenic T cells isolated from the immunized mice at week 1 PI using flow cytometry analysis. The FACS data revealed overall elevation of CD3^+^ total T cell and CD3^+^CD4^+^ T cell subsets in the immunized mice (**Figure 6C**). The significant increase of CD4^+^ T cells in the CD3^+^ gated splenocytes (2.9 ± 1.58 percent) were observed in the immunized mice (**Figure 6B**) compared to those of non-immunized mice (**Figure 6A**) (*P* < 0.05).

### Production of Cytokine mRNA

**FIGURE 7:**
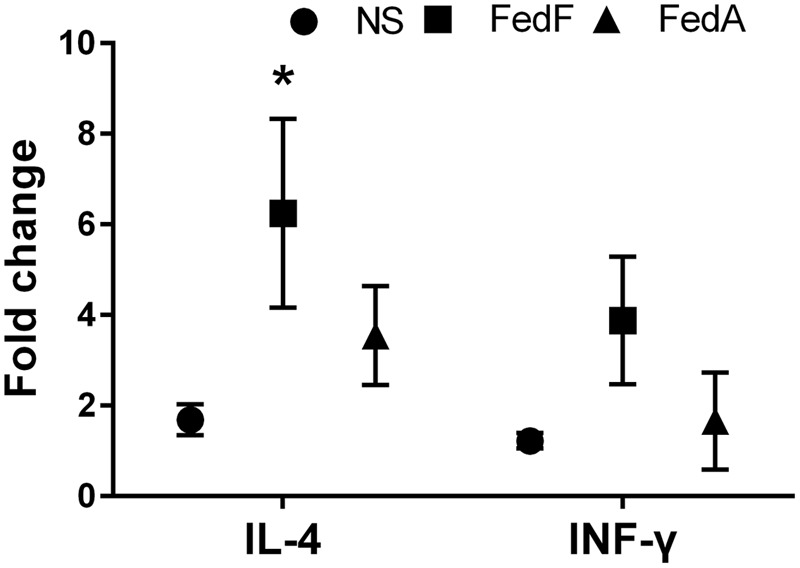
**The cytokine mRNA transcript level in *in vitro* stimulated splenic T cells isolated from mice immunized with JOL1460 and JOL1464, and non-immunized mice using qRT PCR.** The mRNA transcript levels of cytokines were assessed with gene-specific primers, and a relative fold-change was determined by 2^-ΔΔCt^. Each fold-change value represents the mean ± SD of five individual values. NS, non-stimulated cell; ^∗^*P* < 0.05 when the values were compared with those for non-immunized mice.

To evaluate IL-4 and IFN-γ cytokine production modulating the immune responses, we observed fold-changes in mRNA gene expression levels *in vitro* in restimulated splenic T cells collected from the immunized mice using qRT-PCR. A statistically significant augmentation (*P* < 0.05) of IL-4 cytokine was observed in splenocytes restimulated *in vitro* with FedF antigen (**Figure 7**). In the splenocytes restimulated with FedA antigen, the mRNA level of IL-4 cytokine was 3.54 ± 1.09 fold higher than that in the non-immunized group. The level of IFN-γ cytokine was enhanced in both splenocytes stimulated *in vitro* with either FedF or FedA compared to those of the control group.

### Protection Efficacy

**FIGURE 8:**
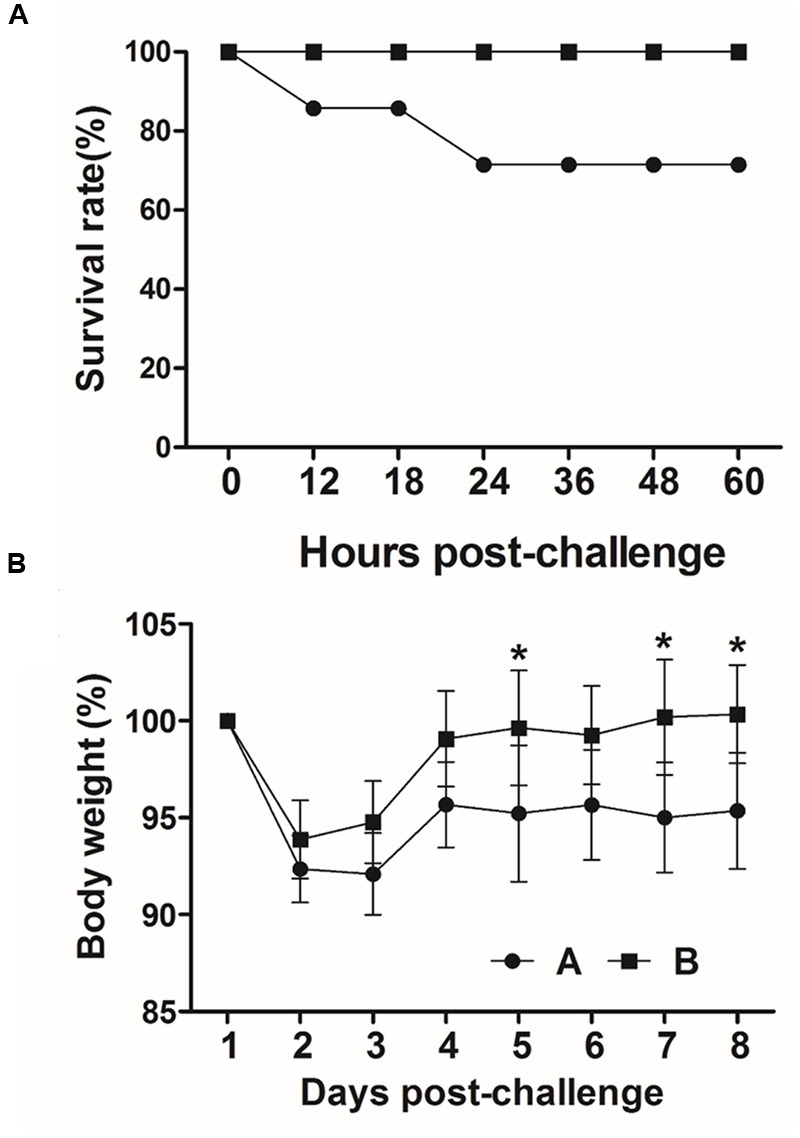
**Protective efficacy of inactivated *Salmonella* strains producing FedF and FedA against a lethal challenge. (A)** Survival rate in immunized and non-immunized mice after challenge. **(B)** Body weight changes (%) in challenged mice. Values are means from seven mice in each group and error bars indicate SD. ^∗^*P* < 0.01 (vs. group A).

To evaluate the protective efficacy conferred by the immunization, all mice in group A (*n* = 7) and B (*n* = 7) were intraperitoneally injected with a lethal dose of the virulent F18^+^STEC strain JOL654 at week 6 PI. The survival rates and weight loss in the mice were monitored in immunized and non-immunized animals for nine days after challenge. In both immunized and non-immunized mice, weight loss was observed until day 2 post challenge. While the weight of immunized animals was fully recovered by day 4 post-challenge, the weight of the non-immunized animals declined until day 9 post-challenge (**Figure 8B**). Particularly, body weight of the non-immunized mice was significantly dropped compared to the immunized mice at day 5, 7, and 8 post-challenge (*P* < 0.01). A clear difference in survival rate was observed between the immunized group and the control group. All immunized mice survived the entire observation period, whereas 28% of the non-immunized mice in the control group died within 24 h after challenge (**Figure 8A**). The clinical signs in the mice such as diarrhea, hunched posture, and hair elections were also observed in non-immunized mice.

## Discussion

Edema disease Infection was initiated by F18 fimbriae, which mediated attachment and colonization of the pathogen to the porcine intestine (Smeds et al., 2003). FedF, F18 fimbrial adhesion, and FedA, the backbone of the F18 fimbrial structure, have been considered as crucial virulence factors of F18^+^ STEC (Imberechts et al., 1992b). In the present study, the genetically engineered *Salmonella* delivery system expressing FedF and FedA was utilized to construct the inactivated vaccine candidates against ED. The resultant strains were designated as JOL1460 and JOL1464, respectively. The plasmid pJHL184 harboring the lysis gene *E* was introduced into the attenuated *S*. Typhimurium strain. The lysis gene *E* placed between the convergent promoter fragments can stringently regulate transcription and expression of the *E* gene in the plasmid pJHL184 (Jawale et al., 2014). Consequently, no viable cells were observed from mid-logarithmic-phase cultures of JOL1460 and JOL1464 after 48 h of lysis, which implied that the protein *E*-mediated lysis, which was adequately controlled by the promoter components, can completely inactivate the candidate strains. Further, the morphological alteration in JOL1460 and JOL1464 ghosts inactivated by the underlying lysis mechanism was identified by SEM study (**Figure 1**). The pores originating from the lysis gene expressions were distinctly visible on the collapsed body with surface folds in the lysed cells.

To demonstrate the capacity of the inactivated JOL1460 and JOL1464 to deliver the heterologous protective antigens, the efficient expression of FedF and FedA fused to *E. coli* outer membrane protein A (OmpA) signal peptide in the strains was validated by immunoblot analysis (**Figure 2**). The *ompA* signal sequence fused in frame efficiently directs secretion of the fusion protein across the periplasmic space (Jawale et al., 2014). Additionally, antibodies specific to FedF and FedA were markedly generated in the immunized mice, which implies that FedF and FedA antigens were sufficiently expressed the inactivated JOL1460 and JOL1464, respectively.

McLamb et al. (2013) reported that weaning stress in piglets negatively affects their mucosal immune response system, resulting in the elevated susceptibility of STEC infection. Thus, mucosal immune responses conferred by immunization play a crucial role in the protection of the post-weaned piglet from ED infection. In this study, sIgA against FedF antigen was significantly increased in the immunized mice (**Figure 2**), which indicates that FedF antigen may be more efficiently presented to the organized lymphoid tissue of the mucosal immune system in the mice (Kraehenbuhl and Neutra, 1992). Further, Externest et al. (2000) showed significant relation among IgA generated in different mucosal effector sites. Meanwhile, the titers of IgG antibody specific to both FedF and FedA were significantly enhanced in the immunized group. Smeds et al. (2001) revealed that serum antibodies specific to FedF efficiently inhibited F18^+^STEC adhesion to porcine enterocyte. Collectively, the results from this study indicate that immunization with JOL1460 and JOL1464 can produce systemic IgG antibodies specific to FedF and FedA, which may impair the mechanism of F18^+^STEC adhesion to the fimbriae-specific receptors on the intestinal enterocyte.

IgG isotypes produced in activated B lymphocytes have been used as parameters to elucidate the type of immune responses elicited by immunization (Coffman et al., 1988). Specifically, the titers of IgG2a and IgG1 isotypes differentiated by cytokines such as IL-2, IL-4, and INF-γ are an indirect measure of the relative contribution of Th-1 and Th-2-related immune responses (Singh et al., 1999). In this study, the level of both IgG2a and IgG1 antibodies specific to FedA and FedF were increased in the immunized mice, which revealed that the JOL1460 and JOL1464 immunization influenced differentiation and proliferation of both Th-1 and Th-2 subsets. Production of IgG2a and IgG1 antibodies specific to FedF was approximately 10 times higher than the antibodies specific to FedA induced in the immunized mice (**Figure 4B**). The substantial increase in IgG isotypes raised against FedF may suggest that FedF antigen expressed in JOL1460 may predominantly affect the immunoglobulin isotype switching in mature B cell lymphocytes by antigen stimulation and relevant cytokine production (Stavnezer et al., 2008). Additionally, the previous report demonstrated that 60–109 amino acid residues of *fedF* are a crucial region for adhesion to porcine intestines (Smeds et al., 2003). This finding suggested that production of a large amount of IgG isotypes against FedF might be associated with the adhesion inhibitory mechanism.

The naïve CD4 T cell subpopulation comprises multipotent precursors that can be differentiated into effector T cells such as Th1, Th2, and Th17 following antigen stimulation (Duffy et al., 2011). In early studies, IL-4 (Swain et al., 1990) and IFN-γ (O’Garra, 1998) were prerequisite autocrine growth factors for Th2 and Th1 cells, respectively. In this study, a slight increase in total splenic T cells and CD4+T cell subsets was shown in *in vitro*-stimulated splenocytes in the mice (**Figure 5**). Concurrently, the expression of IL-4 and IFN-γ mRNA appeared in the *in vitro* stimulated splenic cells (**Figure 7**). This observation suggests that FedF and FedA expressed in JOL1460 and JOL1464, respectively, may stimulate naïve CD4^+^ T cells, which can activate Th1 and Th2 developmental pathways.

Gram-negative bacteria inactivated by the lysis gene *E* have been exploited as a safe vaccine platform against infectious diseases due to their high immunogenicity. Particularly, when the lysed bacteria are employed as the antigen delivery system, intact surface immunostimulatory components are conserved as native forms have an adjuvant property (Riedmann et al., 2007). In the previous study, purified F18 fimbriae protein did not induce significant serum antibody responses (Verdonck et al., 2007), although several variables such as dose, immunization route, and schedule can affect these different results. In our study, the adjuvant effect of the *Salmonella* delivery system may partially account for the significantly elevated titers of serum antibodies specific to FedF and FedA induced by JOL1460 and JOL1464.

Immunization of mice with JOL1460 and JOL1464 conferred effective immunity against intraperitoneal infection of a wild-type of F18ab^+^ STEC. The immunized mice were fully protected from the lethal challenge whereas 28 percent of the mice died in the non-immunized group (**Figure 8**). The antigen-specific protective immunogenicity was elicited by JOL1460 and JOL1464, which indicated that FedF and FedA antigens fused with OmpA signal peptide were adequately secreted and stayed in the inactivated *Salmonella* and consequently were efficiently recognized by the host immune system. Additionally, the serum antibodies derived from the immunization may have the capacity to neutralize the target antigens. Lastly, the development of Th1 and Th2 subsets activated by the immunization may improve the overall immune function.

## Conclusion

The present data indicate that this novel inactivated *Salmonella* delivery system effectively secreted F18+fimbrial proteins, FedF and FedA. Given that the candidate proteins were successfully incorporated into the inactivated *Salmonella* system and led to robust immune responses specific to the target antigens and protective efficacy, the *Salmonella*-producing FedF and FedA may be feasibly utilized as an effective vaccine candidate against porcine edema disease.

## Author Contributions

GW conducted the experiments and was involved in manuscript preparation. JL conceived the study, precipitated in the design of the study and was involved in manuscript preparation.

## Conflict of Interest Statement

The authors declare that the research was conducted in the absence of any commercial or financial relationships that could be construed as a potential conflict of interest.

The reviewer LS and handling Editor declared their shared affiliation and the handling Editor states that the process nevertheless met the standards of a fair and objective review.
